# Serum Angiopoietin-2 level increase differs between polytraumatized patients with and without central nervous system injuries

**DOI:** 10.1038/s41598-023-45688-x

**Published:** 2023-11-07

**Authors:** Lukas L. Negrin, Stefan Hajdu

**Affiliations:** University Department of Orthopedics and Trauma Surgery, Medical University of Vienna, Waehringer Guertel 18-20, 1090 Vienna, Austria

**Keywords:** Biomarkers, Health care, Medical research

## Abstract

Since endothelial cells rapidly release Angiopoietin-2 (Ang-2) in response to vascular injury and inflammatory stimuli, we aimed to investigate if its serum levels increase in polytraumatized patients. Our cohort study evaluated 28 blunt polytrauma survivors (mean age, 38.4 years; median ISS, 34) who were directly admitted to our level I trauma center in 2018. We assessed the serum Ang-2 level at admission and on days 1, 3, 5, 7, and 10 during hospitalization. Ang-2 was released into the circulation immediately after polytrauma. At admission (day 0), it amounted to 8286 ± 5068 pg/mL, three-and-a-half times the reference value of 2337 ± 650 pg/mL assessed in a healthy control group. Subgroup analysis provided a higher mean Ang-2 level in the CNSI group combining all patients suffering a brain or spinal cord injury compared to the non-CNSI group solely on day 0 [11083 ± 5408 pg/mL versus 3963 ± 2062 pg/mL; *p* < 0.001]. Whereas the mean Ang-2 level increased only in the non-CNSI group from day 0 to day 3 (*p* = 0.009), the respective curves showed similar continuous decreases starting with day 3. Multivariate logistic regression analysis revealed an association between the Ang-2 day 0 level and the presence of a CNSI (OR = 1.885; *p* = 0.048). ROC analysis provided a cutoff level of 5352 pg/mL. In our study group, serum Ang-2 levels assessed at admission differed between polytraumatized patients with and without brain or spinal cord injuries. Based on our findings, we consider serum Ang-2 levels an effective biomarker candidate for indicating CNSI in these patients at admission, worthy of further evaluation in large multicenter studies.

## Introduction

Traumatic injuries to the central nervous system (CNSI) are a significant cause of morbidity and mortality in adults and children^1,2^. Managing polytraumatized patients with a concomitant severe traumatic brain injury (TBI) can be challenging because simultaneous surgeries to control hemorrhage and restore perfusion of critically ischemic organs might be necessary while also dealing with the intracranial procedure. Unstable spinal fractures, especially with spinal cord involvement, should be stabilized surgically as soon as possible but have no priority in the acute phase of hemodynamic and respiratory critical patients. Finding a window of opportunity for definitive treatment depends on several confounding factors and often needs a lot of experience without clear guidelines. An early diagnosis is crucial to avoid additional secondary neurological injuries in these patients, who often have a reduced level of consciousness or are under sedative or analgesic medication. Since blood sampling is a standard procedure during initial trauma evaluation, serum biomarker levels might serve as an additional tool to computed tomography as the golden standard.

Injuries to the CNSI result from direct or indirect mechanical forces on the brain or the spinal cord, damaging cells and initiating a complex secondary injury cascade^3–5^. The blood–brain barrier shields the brain from toxic substances in the blood; it supplies brain tissues with nutrients and filters harmful compounds from the brain back to the bloodstream^6^. The blood-spinal cord barrier mediates the exchange of substances between the blood and the spinal cord^7^. Both barriers are similarly structured as their first layers are formed by tightly connected endothelial cells lining the capillaries penetrating the brain or spinal cord^7–10^. Except for tumor cells^11,12^, vascular endothelial cells have been identified as the primary source of Angiopoietin-2 (Ang-2)^13^. Ang-2 is a secreted glycoprotein growth factor^14,15^. It is essential in modulating angiogenesis and vascular homeostasis^16^ and is a potential marker for endothelial activation and dysfunction^17^. Ang‐2 can act in an autocrine manner on endothelial cells. It can destabilize blood vessels, enhance vascular leakage, induce vascular regression, and prime the endothelium to respond to angiogenetic and inflammatory cytokines^18^. Upon stimulation, innate immune cells can react with increased adhesion to the vessel wall^13^. Moreover, Ang‐2 can act in a paracrine manner on leukocytes, particularly myeloid lineage cells^19^.

Laboratory tests on rats have revealed an increase in Ang-2 levels in the early phase after inflicting brain injury^20^ and a persistent rise (up to ten weeks) after causing spinal cord injuries^21^. In humans, plasma Ang-2 levels were increased shortly after traumatic spinal cord^22^ and pediatric traumatic brain injury^23^. Numerous papers have already been published focusing on Ang-2 levels and ARDS development in non-traumatic settings; some are mentioned as examples. Plasma Ang-2 levels were significantly increased in patients with ARDS^24^ and predicted ARDS onset in critically ill patients^25^. In this patient population, high serum Ang-2 levels were also associated with poor outcomes^26^. According to a meta-analysis, more elevated serum Ang-2 levels at baseline were independently associated with a 56% increase in mortality risk in patients with ARDS^27^. Moreover, serum Ang-2 levels were higher in patients suffering from pneumonia than in healthy individuals and predicted mortality and length of hospital stay^28^.

In light of these findings, we hypothesized that (1) serum Ang-2 levels significantly increase after polytrauma, remaining elevated for a couple of days, (2) serum Ang-2 secretion is more pronounced in polytraumatized patients suffering CNSI, and (3) serum Ang-2 levels may indicate traumatic brain or spinal cord injuries in polytraumatized patients.

## Methods

### Ethical statements

This study was approved by the institutional review board of the Medical University of Vienna (Austria) (vote 1617/2018) and was conducted in accordance with the Declaration of Helsinki and applicable local regulations. Written informed consent was obtained from all patients.

### Patients

For our cohort study, we prospectively enrolled 28 blunt polytrauma survivors who were directly admitted to our level I trauma center in 2018 and were transferred to the intensive care unit after initial treatment. Inclusion criteria were a minimum age of 18 and an Injury Severity Score (ISS) ≥ 16. We excluded patients with known malignancies and chronic inflammatory lung diseases. Ten healthy adults who responded to our call for volunteers were combined into the control group. We retrospectively evaluated our results, adhering to the STROBE Statement. The corresponding checklist is presented as a supplemental file. In computed tomography scans, patients were assigned to the CNSI group if there was evidence of structural brain or spinal cord damage by hemorrhage, swelling, compression, or fractures.

### Biomarker assessment

For initial Ang-2 level measurement, 8 mL venous blood (using Vacuette^®^ tubes; Greiner Bio-One International) was withdrawn from each patient within 10 min after arrival at our trauma center and centrifuged at 3000 g for 15 min at room temperature immediately after that. The serum was removed and stored at − 80 °C until assayed promptly. Blood samples were retaken on days 1, 3, 5, 7, and 10 during hospitalization as long as the patient consented. For biomarker analyses, we used Luminex multi-analyte technology (R & D systems Magnetic Luminex^®^ Screening Assay—human Premixed Multi-Analyze Kit Number LXSAHM). We performed all measurements in technical duplicates and calculated the respective mean values. We informed our patients about blood sampling at the earliest time point possible. In case they refused written consent, no further blood samples were taken, and the already acquired material was destroyed if requested by the patient. However, only one blood sample was drawn in the control group.

### Statistical analysis

Statistical analysis was performed using the statistical software IBM SPSS Statistics 29. The Kolmogorov–Smirnov Test was applied to test for normality. Continuous data are presented as mean ± standard deviation if normally distributed, otherwise as median and interquartile range in round brackets. Absolute frequencies and percentages characterize categorical data. Since the biomarker levels assessed on days 1, 3, 5, 7, and 10 were normally distributed, and the day 0 level did not include outliers, we used the mean value and error bars for the standard error of the mean (SEM) to graphically display differences in mean Ang-2 levels between the CNSI and the non-CNSI group.

According to the distribution, independent-sample two-tailed t-tests or Mann–Whitney-U-Tests were calculated to reveal intergroup differences. In contrast, dependent two-tailed t-tests were conducted for paired samples of Ang-2 levels. Categorical data were compared through the Chi-Square Test. To reveal associations between the Ang-2 level at admission and age, ISS, and the assessed clinical parameters, we calculated Spearman’s correlation coefficients. Univariate and multivariate binary logistic regression analysis was performed to quantify the strength of the association between initial Ang-2 levels and the presence of a CNSI. The odds ratio (OR) was presented with a 95% confidence interval (CI). A receiver operating characteristic (ROC) curve was created, and the area under the curve (AUC) was computed. The cutoff value was determined by the maximum sum of sensitivity and specificity^29^. In general, a *p*-value of < 0.05 was considered significant.

## Results

According to our inclusion and exclusion criteria, 28 polytrauma survivors (21 males and seven females) with a mean age of 38.4 ± 19.6 years and a median Injury Severity Score (ISS) of 34 (30–41) formed our study group. Causes of polytrauma were car crashes (11), vehicle-, streetcar- or train-pedestrian collisions (9, including three suicide attempts), falls from a height ≥ 2 m (6, including one suicide attempt) as well as one overrun by a wheel loader and one violent crime. Two polytrauma victims withdrew their consent for a further blood draw after their transfer from the ICU to a regular ward on day 8. One patient was discharged to another hospital on day 9. Therefore, only 25 samples were available for day 10. However, complete demographic data and clinical parameters could be collected for all victims. Eleven patients developed acute respiratory syndrome (ARDS), 16 sustained pneumonia (six suffered both complications), and nine underwent secondary surgery. Extracorporeal membrane oxygenation (ECMO) was applied to two and hemofiltration to one patient. Median ventilation time was calculated to be 10.5 (7.3–16) days, and the mean length of stay at the ICU and the hospital amounted to 22.3 ± 12.0 days and 45.5 ± 25.7 days, respectively.

Table 1 presents each patient's initial Ang-2 level and ISS, AIS_Head_, AIS_Face_, AIS_Thorax_, AIS_Abdomen_, AIS_Spine_, and AIS_Extremities_ (AIS, Abbreviated Injury Scale) values. Table 2 compares the CNSI and the non-CNSI groups based on demographic data and clinical parameters assessed at admission. We calculated only a weak correlation between the Ang-2 day 0 level and the base excess (ρ = 0.466; *p* < 0.05) and the thrombocytes (ρ = 0.382; *p* < 0.05), respectively.

**Table 1:** Overall injury severity and severity of the single injuries.

Patient number	Group	Ang-2 level day 0 pg/mL	ISS	AIS					
				Head	Face	Thorax	Abdomen	Spine	Extremities
1	CNSI	12,862	29	4	3	2	3	0	2
2	CNSI	9606	30	5	1	1	0	0	2
3	CNSI	11,284	26	5	1	0	0	0	0
4	Non-CNSI	2459	43	0	3	5	0	0	3
5	Non-CNSI	1195	34	0	3	4	0	0	3
6	CNSI	14,919	34	5	3	0	0	0	3
7	CNSI	23,564	34	0	0	2	3	4	3
8	Non-CNSI	1931	50	0	3	5	0	0	4
9	Non-CNSI	4828	34	0	0	3	0	0	5
10	Non-CNSI	4961	21	0	4	0	1	0	2
11	CNSI	16,672	34	0	2	3	4	3	2
12	Non-CNSI	4531	33	0	0	5	2	0	2
13	CNSI	9407	33	2	0	5	0	0	2
14	CNSI	15,309	50	0	0	5	0	5	0
15	CNSI	8771	41	5	0	0	0	4	0
16	Non-CNSI	4664	41	0	3	4	4	0	0
17	CNSI	4050	45	5	2	4	0	0	2
18	Non-CNSI	8585	22	0	0	3	2	0	3
19	CNSI	7253	22	3	3	3	2	0	0
20	CNSI	4328	48	4	2	4	4	0	2
21	CNSI	16,819	45	4	0	5	0	0	2
22	CNSI	14,666	34	0	0	3	3	4	3
23	CNSI	8857	27	3	1	3	0	0	3
24	CNSI	5743	34	3	2	4	3	0	0
25	Non-CNSI	3844	34	0	0	4	3	2	3
26	Non-CNSI	4653	41	0	0	4	4	2	3
27	CNSI	4309	30	2	1	1	0	5	0
28	Non-CNSI	1946	35	0	0	5	3	0	1

**Table 2:** Demographic data and clinical parameters assessed at admission.

	Non-CNSI group	CNSI group	*p*-value
Number of patients (n)	11	17	
Age (years)	37.9 ± 19.5	38.7 ± 20.3	0.459
Males (n)	8 (72.7%)	13 (76.5%)	0.823
ISS	34 (33–41)	34 (29.5–43)	0.711
AIS_Head_ ≥ 3 (n)	0 (0%)	11 (64.7%)	** < 0.001**
AIS_Face_ ≥ 3 (n)	5 (45.5%)	3 (17.6%)	0.200
AIS_Thorax_ ≥ 3 (n)	10 (90.9%)	10 (58.8%)	0.099
AIS_Abdomen_ ≥ 3 (n)	4 (36,4%)	6 (35.3%)	0.954
AIS_Spine_ ≥ 3 (n)	0 (0%)	6 (35.3%)	**0.026**
AIS_Extremities_ ≥ 3 (n)	7 (63.6%)	4 (23.5%)	0.053
ARDS (n)	7 (63.6%)	4 (57.1%)	0.053
Pneumonie (n)	5 (45.5%)	7 (41.2%)	0.823
ICU (days)	19.8 ± 6.1	23.9 ± 14.7	0.321
LOS (days)	38.7 ± 25.4	49.8 ± 25.7	0.237
Ventilation (days)	10 (9–12)	11 (5–27)	0.711
Secondary surgery (n)	5 (45.5%)	4 (23.5%)	0.225
Ang-2 day 0 level	4531 (1946–4828) pg/mL	9606 (6498–15,114) pg/mL	< 0.001
Lactate	3.0 (2.1–5.3) mmol/L	2.4 (1.5–4.0]) mmol/L	0.175
Base excess	− 6.2 ((− 14.1)–(− 4.0)) mmol/L	− 3.1 ((− 5.3)–(− 2.3)) mmol/L	**0.002**
Shock index	0.9 ± 0.2	0.8 ± 0.2	**0.045**
Hemoglobin	10.3 ± 2.5 g/dL	11.7 ± 2.2 g/dL	0.150
Thrombocytes	170 ± 71 /nL	200 ± 63 /nL	0.259
Fibrinogen	162 ± 51 mg/dL	220 ± 97 mg/dL	0.091
NT-ProBNP	77 ± 40 pg/mL	91 ± 21 pg/mL	0.333
Creatine Kinase	466 (305–931) ng/mL	361 (235–840) ng/mL	0.517
Thrombin time	16 (15–18) seconds	14 (14–17) seconds	0.195
Activated partial thromboplastin	39 (33–49) seconds	36 (32–38) seconds	0.359

Significant values are in bold.

**Figure 1:**
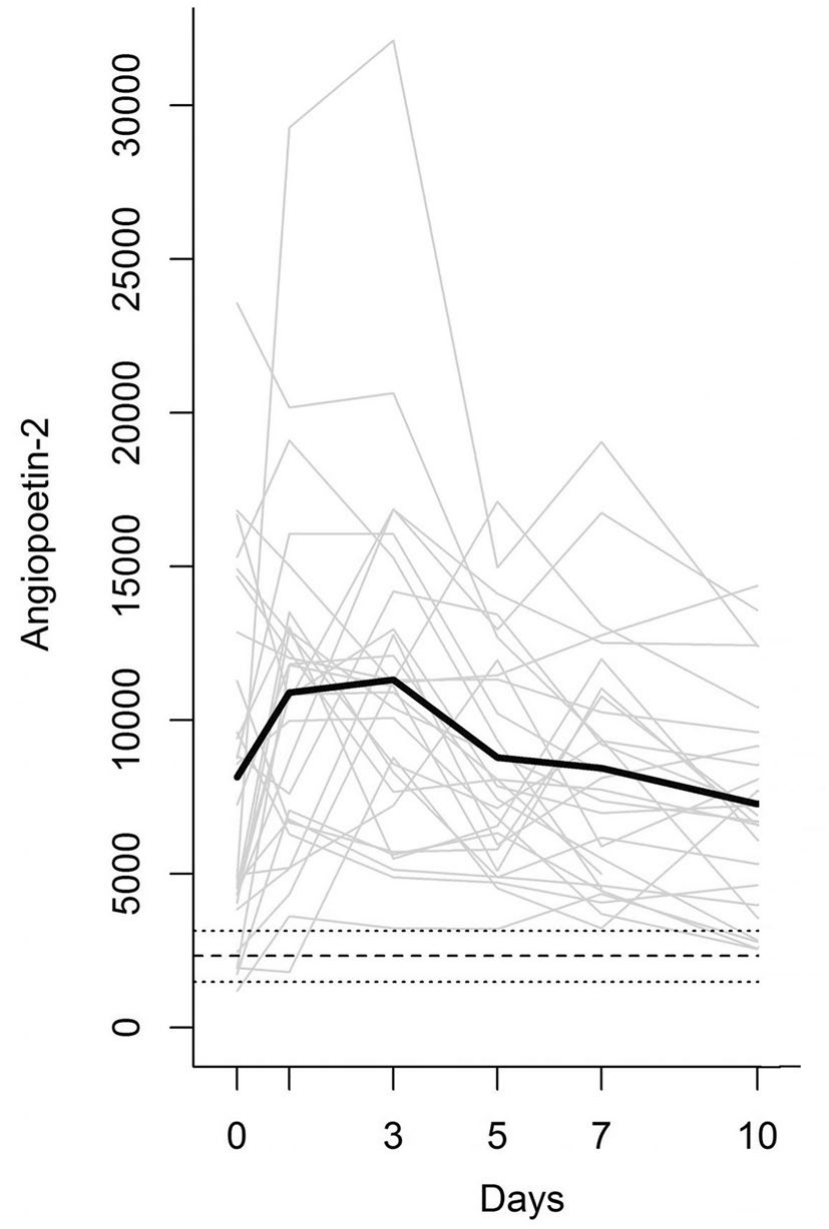
Individual Ang-2 levels (grey lines) and mean Ang-2 level (bold black line) in the study group. The dotted and the two dashed parallel lines represent the mean, minimum, and maximum levels provided by healthy controls.

Figure 1 displays the individual Ang-2 levels and the mean Ang-2 level within the first ten posttraumatic days and the mean, minimum, and maximum levels provided by five male and five female healthy controls with a mean age of 38.7 ± 20.5 years. The mean Ang-2 level increased from day 0 to day 3 (*p* = 0.018) and decreased from day 3 to day 10 (*p* = 0.001), not differing between day 0 and day 10 (*p* = 0.715). At admission, it amounted to 8286 ± 5068 pg/mL, three-and-a-half times the reference value of 2337 ± 650 pg/mL assessed in the healthy control group. In contrast, the median Ang-2 level exceeded 3.7 fold of the corresponding reference value [9606 (6498–15,114) pg/mL versus 2576 (1616–2866) pg/mL] at this time. Mean and median Ang-2 day 0 levels did not significantly differ between males and females.

**Figure 2:**
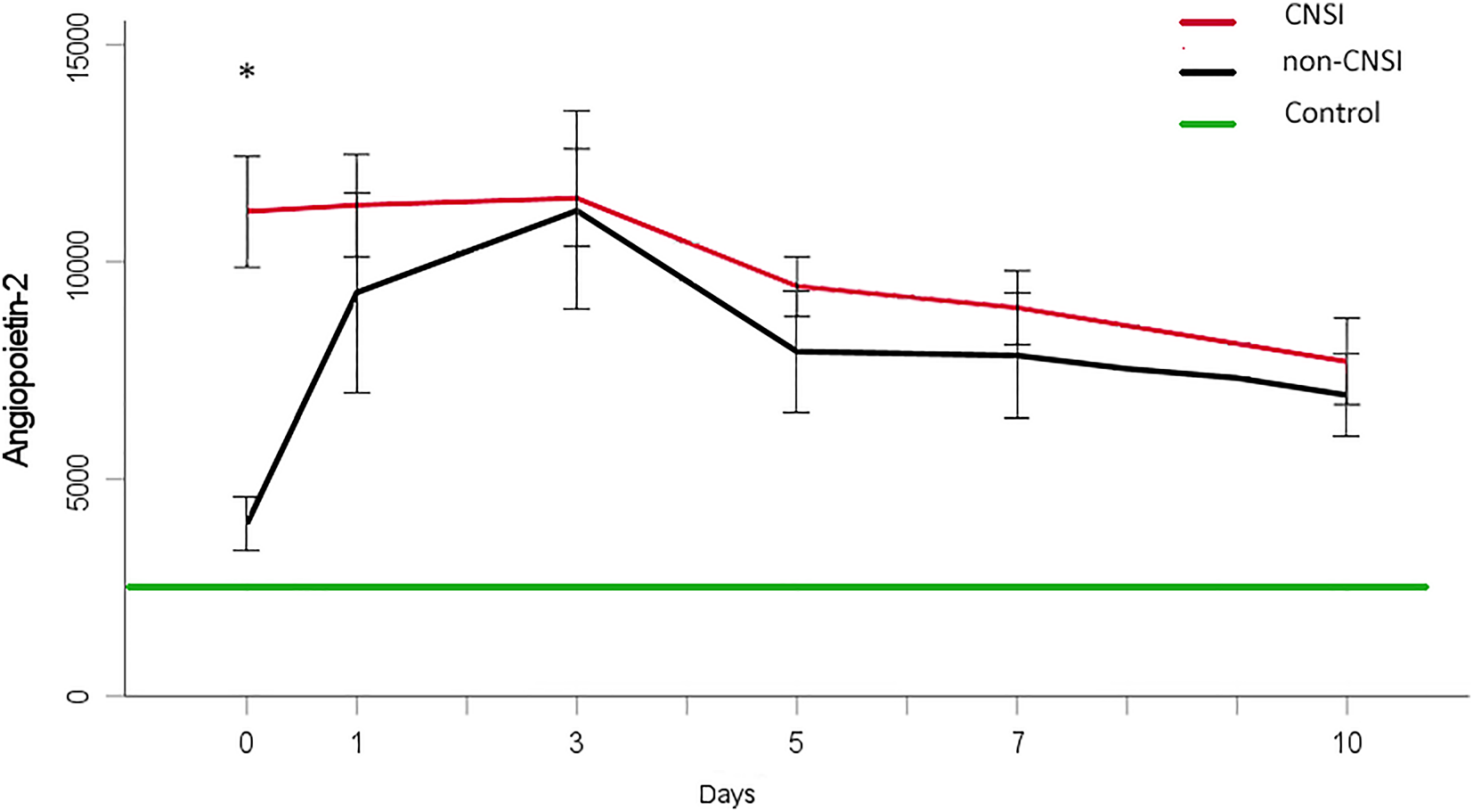
Mean Ang-2 levels in the CNSI-group (red line), comprising all individuals suffering injuries to the central nervous system and in the non-CNSI-group (black line), which combines all individuals without brain and spinal cord injuries. Error bars display SEM, * indicates a significant difference between the groups. The green line represents the mean value provided by healthy controls.

Figure 2 presents the time course of the mean Ang-2 level and the corresponding SEM bars in the CNSI and non-CNSI groups. Both intergroup comparison and overlapping of the error bars only revealed a significant difference in mean Ang-2 levels at admission (11,083 ± 5408 pg/mL versus 3963 ± 2062 pg/mL; *p* < 0.001). Remarkably, there was only a significant increase between the mean day 0 and day 3 levels in the non-CNSI group (*p* = 0.009). However, the respective curves showed similar steady declines beginning with day 3 but not falling below the reference value provided by the control group during the observation period.

Since the day 0 levels ranged from 1159 to 23,564 pg/mL, we defined its thousandths as the independent variable of the binary logistic regression analysis for the presence of a CNSI, resulting in an OR = 1.879; 95% CI (1.129–3.128); *p* = 0.015. Table 2 shows that the base excess and the shock index at admission significantly differed between the non-CNSI and the CNSI group. Therefore, we adjusted our model for these parameters. Nevertheless, multivariate binary logistic regression analysis identified solely the Ang-2 level at admission as an indicator of a CNSI presence (OR = 1.885, 95% CI (1.058–3.360); *p* = 0.048).

**Figure 3:**
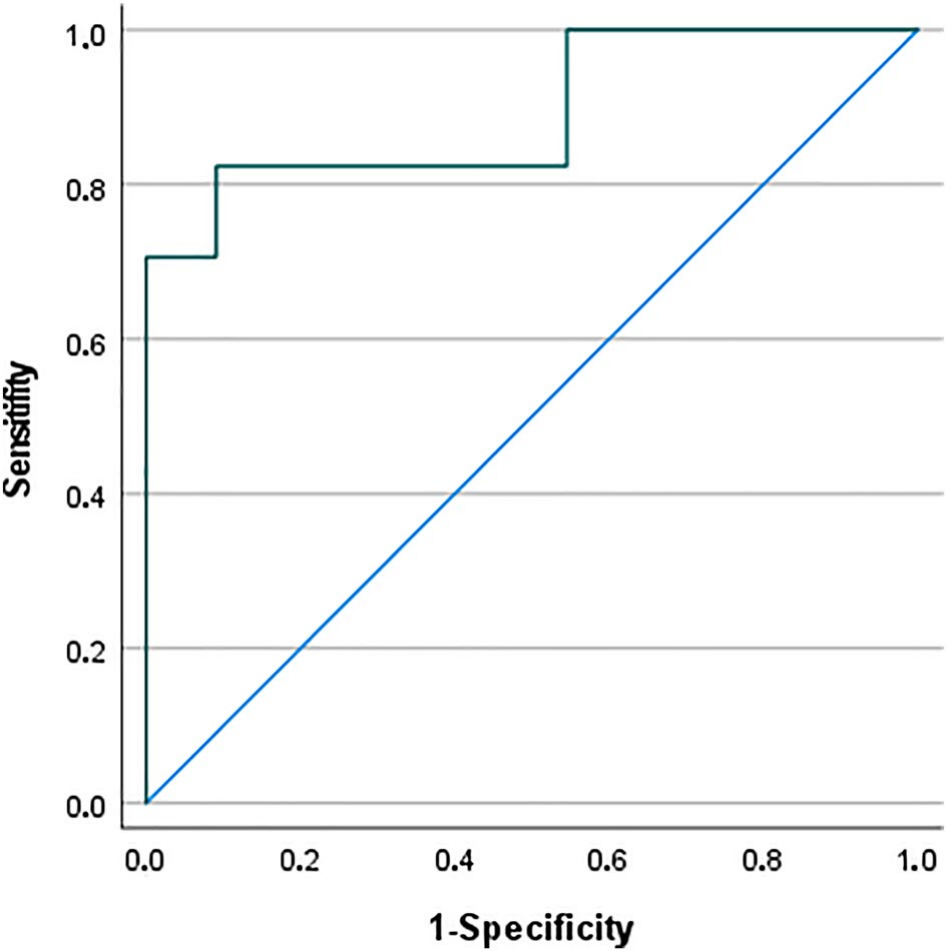
ROC curve of the serum Ang-2 level at admission and the presence of a CNSI in polytraumatized patients.

Finally, the ROC curve of the serum Ang-2 level at admission and the existence of a CNSI in polytraumatized patients is presented in Fig. 3. ROC analysis provided an AUC = 0.893 with a cutoff value of 5352 pg/mL (sensitivity, 82.4%; specificity, 90.9%).

## Discussion

In polytraumatized patients, Ang-2 was released into the circulation immediately after the injuries occurred, resulting in a significantly higher mean serum Ang-2 level in the CNSI group than in the non-CNSI group at admission, with both mean values not falling below the reference value provided by the healthy controls throughout the observation period.

Two distinct mechanisms of Ang-2 secretion from endothelial cells have been identified^13^. Over time, a slow but continuous increase in Ang-2 level has been proven in a culture medium without any exogenous stimulus, indicating that Ang-2 molecules are derived from de novo synthesis (constitutive secretion). Contrarily, it has been shown that exogenous stimuli induce a dose-dependent increase in Ang-2 level in a culture medium within less than one minute (stimulated secretion)^13^. Ang-2 is mainly stored in the cytoplasmic secretory granules of the endothelial cells called Weibel-Palade bodies^18,30^, featuring a half-life of more than 18 h^30^. It is selectively released after exogenous endothelial activation (including inflammatory stimuli, shear stress, or vascular injury), which induces exocytosis of the Weibel-Palade bodies^30,31^, causing an extraordinarily rapid and sharp rise in Ang-2 level^13,18^, followed by a gradual decrease^13^, and recovering within a few hours after release^30^.

Ang-2 is only weakly expressed by the quiescent endothelium through constitutive secretion^22^. Due to the severe tissue damage caused by several injuries, a local and systemic inflammatory response was induced after polytrauma that provoked a rapid release of Ang-2 into circulation through stimulated secretion after endothelial activation. As Table 1 shows, our patients suffered a variety of injury patterns. Since the distribution of the Weibel-Palade bodies along the vascular tree is heterogeneous^29^ and their content differs between vascular beds^24^, they might release varying amounts of Ang-2 due to various storage capacities immediately after the polytrauma occurs, resulting in a wide range of the day 0 levels. As the Weibel-Palade bodies recover within hours, repetitively releasing many Ang-2 molecules, and the reduction in Ang-2 concentration occurs slowly, Ang-2 levels remained elevated during the entire study period.

Several studies have focused on Ang-2 in a traumatic setting. The observed increase in Ang-2 levels after polytrauma in our patient population complies with results already presented in the literature. Plasma Ang-2 levels rose soon after the trauma and correlated with the ISS^32^. Plasma Ang-2 levels were elevated in trauma patients with a median ISS of 34 with acute lung injury or ARDS^33^. Moreover, they were significantly higher in patients presenting with an ISS ≥ 16 than those with an ISS < 16 and in fatalities compared to survivors^34^. A plasma biomarker panel of Ang-2 and RAGE diagnosed ARDS in patients suffering severe traumatic injuries^35^. Serum Ang-2 levels were increased and were related to a bad prognosis in multiple injured patients (ISS ≥ 25) developing septic complications^36^. In children suffering mild, moderate, and severe TBI, no relationship between plasma Ang-2 levels and TBI severity, age, and gender could be detected^23^. We found no correlation between serum Ang-2 day 0 levels and age and no gender-specific differences in Ang-2 day 0 levels either. Levels of Ang-2 in cerebrospinal fluid have already been identified as sensitive and specific biomarker candidates to indicate TBI^37^. In patients with traumatic spinal cord injury, plasma Ang2 levels increased after the injury occurred^22^. Tranexamic acid has been shown to inhibit early upregulation of Ang-2 in patients with moderate-to-severe TBI^38^.

As displayed in Table 2, the CNSI and the non-CNSI group did not significantly differ in injury severity assessed by ISS and AIS_Face_, AIS_Thorax_, AIS_Abdomen_, AIS_Extremities_ values, and the presence of ARDS and pneumonia. Therefore, their contribution to the amount of Ang-2 released in circulation immediately after polytrauma can be considered approximately the same in both groups. This conclusion suggests that brain and spinal cord injuries caused higher Ang-2 levels in the CNSI group.

The dichotomization of our patients according to the presence of a CNSI is displayed in Fig. 2. It shows a steep rise in the Ang-2 level in the CNSI group immediately after the polytrauma. Our results are partly in line with those found in rat models. Ang-2 expression began to increase after subarachnoid hemorrhage had been induced by an endovascular perforation technique^39^. Contrarily, the infliction of a spinal cord injury resulted in a significant decrease in the Ang-2 protein levels 24 h afterward at the lesion site^21^.

Similar mean maximum levels in the CNSI and the non-CNSI group suggest that circulating Ang-2 is directly proportional to the number of damaged endothelial cells independent of their location. The different periods until the concentration reaches its mean top value qualify initial serum Ang-2 levels to indicate the presence of a CNSI in a polytrauma setting. A higher storage capacity of Weibel-Palade bodies located in the central nervous system compared to other body regions or another release mechanism that still needs to be explored might be the reason for this finding. According to the AUC of 0.893, serum Ang-2 levels assessed at admission can accurately indicate polytraumatized patients with concomitant CNSI, providing a false-positive rate of 17.6% and a false-negative rate of 9.1% when setting the cutoff value to 5352 pg/mL. An increase in the Ang-2 level by 1000 pg/mL increases the odds of a CNSI’s presence by 88.5% (OR = 1.885).

Revealing significantly higher mean Ang-2 levels in polytrauma victims suffering a CNSI compared to those without a CNSI if assessed at admission, we identified Ang-2 as a promising biomarker candidate for indicating acute brain and spinal cord damage in this study population, worthy of further evaluation. We hope our findings will be verified and validated using independent sample cohorts, ideally collected in a large multicenter study due to existing clinical and biological variability, to determine if sufficient evidence exists for its potential clinical utility. Even though serum Ang-2 levels will be rated clinically relevant at admission, they will be implemented in polytrauma care only if kits are available to perform the tests in the resuscitation room or an affiliated laboratory, providing reliable results within minutes. Finally, additional biomarkers that assess inflammation and endothelial injury should be included in the multicenter study since a biomarker panel may offer higher sensitivity and specificity for diagnosis and prognosis than Ang-2 alone.

Limitations of our study include the fact that we did not perform an a priori power analysis to estimate the minimum sample size. Instead, we relied on the patient numbers of already published pilot studies^40–44^. Furthermore, the number of biomarker samples was determined by the hospital discharge date or the patient's willingness, thus resulting in a complete data set of biomarker levels only for the first seven posttraumatic days. Finally, the study population was limited to a single level I trauma center.

### Supplementary Information

Supplementary Information.

## Data Availability

The data used to support the findings of this study are included in the article.
